# Concerns about anti-angiogenic treatment in patients with glioblastoma multiforme

**DOI:** 10.1186/1471-2407-9-444

**Published:** 2009-12-16

**Authors:** Joost JC Verhoeff, Olaf van Tellingen, An Claes, Lukas JA Stalpers, Myra E van Linde, Dirk J Richel, William PJ Leenders, Wouter R van Furth

**Affiliations:** 1 Department of Radiation Oncology, Academic Medical Center, Meibergdreef 9, 1105 AZ Amsterdam, The Netherlands; 2 Department of Diagnostic Oncology, Netherlands Cancer Institute, Plesmanlaan 121, 1066 CX Amsterdam, The Netherlands; 3 Department of Pathology, Radboud University Nijmegen Medical Centre, Geert Grooteplein-Zuid 10, 6525 GA Nijmegen, The Netherlands; 4 Department of Medical Oncology, Academic Medical Center, Meibergdreef 9, 1105 AZ Amsterdam, The Netherlands; 5 Neurosurgical Center Amsterdam, Academic Medical Center, Meibergdreef 9, 1105 AZ Amsterdam, The Netherlands

## Abstract

**Background:**

The relevance of angiogenesis inhibition in the treatment of glioblastoma multiforme (GBM) should be considered in the unique context of malignant brain tumours. Although patients benefit greatly from reduced cerebral oedema and intracranial pressure, this important clinical improvement on its own may not be considered as an anti-tumour effect.

**Discussion:**

GBM can be roughly separated into an angiogenic component, and an invasive or migratory component. Although this latter component seems inert to anti-angiogenic therapy, it is of major importance for disease progression and survival. We reviewed all relevant literature. Published data support that clinical symptoms are tempered by anti-angiogenic treatment, but that tumour invasion continues. Unfortunately, current imaging modalities are affected by anti-angiogenic treatment too, making it even harder to define tumour margins. To illustrate this we present MRI, biopsy and autopsy specimens from bevacizumab-treated patients.

Moreover, while treatment of other tumour types may be improved by combining chemotherapy with anti-angiogenic drugs, inhibiting angiogenesis in GBM may antagonise the efficacy of chemotherapeutic drugs by normalising the blood-brain barrier function.

**Summary:**

Although angiogenesis inhibition is of considerable value for symptom reduction in GBM patients, lack of proof of a true anti-tumour effect raises concerns about the place of this type of therapy in the treatment of GBM.

## Background

The brain is the highest organised and most complex organ of the body. Some unique features of the brain that have an impact on the biology of brain tumours include extensive three-dimensional structuring with gray and white matter [1], and high density of blood capillaries making it a well-perfused organ; however, a blood-brain barrier (BBB) very selectively regulates the penetration of substances into the brain [2]. With respect to this specific micro-environment, brain cancer, in particular high-grade astrocytoma (malignant glioma, glioblastoma multiforme, GBM) differs from many other cancer types. Whereas this disease usually manifests itself as a focal lesion with central necrosis surrounded by an angiogenic tumour rim (one of the characteristics of GBM), this tumour also invades the surrounding extracellular matrix, using both white matter tracts and blood vessels as substrate [3,4]. Consequently, the presence of migrating glioma cells in brain parenchyma relatively far away from the tumour core is common, complicating curative surgery and radiotherapy. The exact molecular and cellular mechanisms behind cell migration in glioma remain to be elucidated [5].

Despite attempts to improve treatment results over the last 30 years, GBM remains a highly fatal and devastating disease; a cure is extremely rare, even with aggressive treatment [6,7]. The current standard of care is surgical resection to a maximal feasible extent, followed by radiotherapy and systemic temozolomide (TMZ) chemotherapy. The latter has a modest effect on outcome, with a median overall survival of 14.6 months versus 12.1 months for radiotherapy alone [8]. Consequently, with so few treatment options available, the latest regimens designed for treatment of other cancers are currently also tested for GBM. These new agents include angiogenesis inhibitors, in particular bevacizumab (Avastin^®^). Combined with chemotherapy, this monoclonal antibody against VEGF-A is FDA approved for the treatment of advanced non small-cell lung cancer, advanced breast cancer and advanced colorectal cancer, improving overall survival in these patients compared with chemotherapy alone [9-13]. Indeed, results of phase II non-randomised trials with angiogenesis inhibitors for recurrent GBM seem promising, with substantial improvement of apparent median progression-free survival (PFS) and 6-months PFS6 compared with historical controls [14-17]. Due to a lack of large phase III studies, reliable data on overall survival prolongation are not available. Yet, based on data from phase II studies, bevacizumab received accelerated FDA approval for treatment of recurrent high-grade astrocytomas in May 2009.

Despite these apparent improvements it should also be realised that the characteristic tumour micro-environment in brain may have a negative impact on the efficacy of such therapies, and may even be responsible for overestimation of efficacy. We outline below how the unique features of GBM may provide this type of tumour with an 'escape mechanism' from anti-angiogenic treatment, and present clinical and histological data that confirm results from preclinical work on orthotopic glioma xenografts. While angiogenesis inhibitors in combination with chemotherapies may effectively inhibit tumour cell proliferation in other tumour types, the brain micro-environment allows furtive invasion and proliferation in case of GBM.

## Discussion

### Clinical experiences

**Figure 1:**
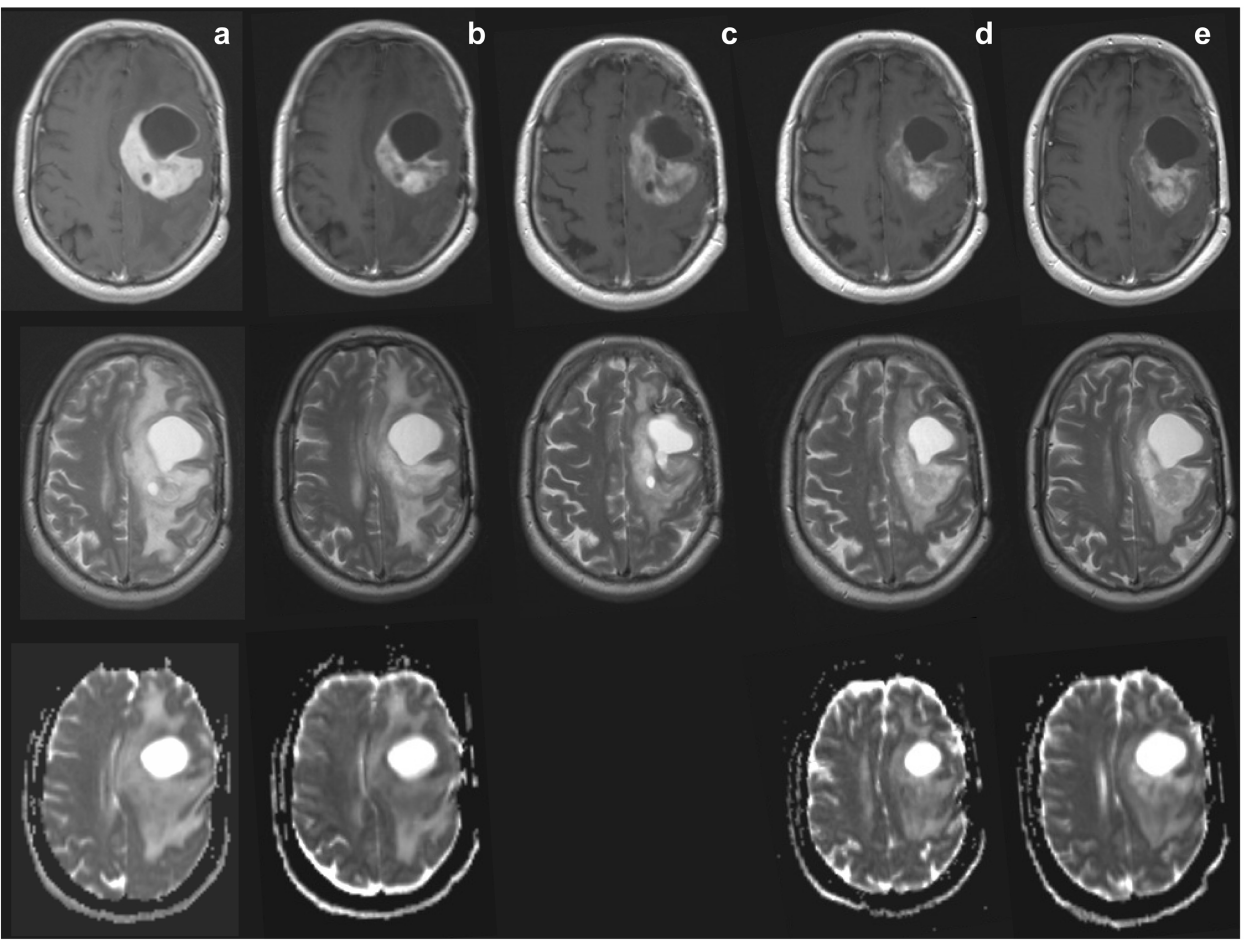
**MRI scans of recurrent GBM treated with bevacizumab**. MRI scans of a typical patient with recurrent GBM, treated with bevacizumab 10 mg/kg every 3 weeks plus daily temozolomide 50 mg/m^2^. Top row T1; middle row T2; bottom row ADC (apparent diffusion coefficient. ADC c is lacking). Column (**A**) scans pre-treatment, showing cystic and tumour component, large midline shift, and large vasogenic oedema. Column (**B**) 3 days after start, showing reduced contrast enhancement, and slightly reduced midline shift. Column (**C**) 21 days after start, showing reduced contrast enhancement but a larger size (no progression based on Macdonald criteria), reduced midline shift, and reduced oedema. Column (**D**) 88 days after start, showing decreased size of tumour and cystic component, stable reduction of contrast enhancement, normalised midline shift, and slight increase of oedema. Column (**E**) 188 days under treatment, showing increased tumour size and cystic component, increased midline shift, and increased oedema (also in the other hemisphere).

One of the aggravating symptoms of high-grade astrocytomas is increased intracranial pressure, a direct result of oedema caused by leaky tumour vasculature. Corticosteroids are used to alleviate these symptoms [18]. Angiogenesis inhibitors are also effective in reducing these symptoms, or in reducing the need for corticosteroid treatment in recurrent GBM patients [19-26]. This rapid effect of anti-VEGF therapy is suggested to be due to a normalization of vascular permeability resulting in a reduction of peritumoural oedema and intracranial pressure, and is even more active than corticosteroid therapy alone (Figure 1A-C) [21,24].

However, the question remains whether angiogenesis inhibitors represent a true anti-GBM regimen. Non-randomised phase II trials in recurrent GBM patients demonstrated high response rates ranging from 20% with single agent TMZ [27], to 30% with single agent AZD2171 (Cediranib, a small molecule VEGFR inhibitor) [24], 20% with single agent bevacizumab, and even to 57% when bevacizumab was combined with irinotecan [14-17,28]. These studies also demonstrated longer median PFS (14-24 weeks), and 6-months PFS (17%-46%), compared with historical controls treated with cytotoxic chemotherapy (6-months PFS mostly <30%). Although some have reported relatively long median survival (34-42 weeks) [14,16,28], Norden et al. [29] demonstrated a longer median PFS (8 vs. 22 weeks), but not median survival (39 vs. 37 weeks), of recurrent GBM patients treated with anti-angiogenic therapy. The suggested efficacy of anti-angiogenic drugs in patients with recurrent GBM is based on non-randomised trials with PFS as the main study endpoint.

Determination of the precise time at which GBM progression occurs is challenging. The often robust and sustained changes on MRI by anti-angiogenic drugs may render subsequent MRI scans difficult to interpret [30]. Therefore, the response rate and PFS may not be the optimal endpoints in phase II studies of anti-angiogenic therapy in patients with recurrent GBM. Because of the potential selection bias in non-randomised studies, and concerns about PFS as study endpoint, we should interpret data from such studies with caution [31]. Randomised trials with survival as primary endpoint are necessary to determine the role of angiogenesis inhibitors in the treatment of patients with recurrent GBM.

A problem with the current enthusiasm for angiogenesis inhibition in GBM is that tumour response evaluations are based on cross sectional contrast enhancing tumour areas [32]. Because anti-angiogenic therapy directly interferes with vessel permeability and gadolinium-DTPA (Gd-DTPA) uptake in the tumours, response evaluations based on contrast enhancing areas are suboptimal for monitoring the effects of anti-angiogenic therapy [30]. In addition, the current methods of imaging are not adequate to identify tumour boundaries, the areas where tumour growth and infiltration takes place. Indeed, also in mouse models of orthotopic glioma, tumour visibility on Gd-DTPA-enhanced MRI scans show an impressive decrease after anti-angiogenic treatment. However, in these models treatment does not affect tumour growth, but merely shifts tumour progression from an expansive to an invasive, angiogenesis-independent phenotype [33,34]. Therefore, also in patients with GBM, the impressive decreases of contrast enhancement in these tumours on anti-angiogenic treatment (Figure 1A-E) are not necessarily synonymous with anti-tumour effects.

In the 1990s, MRI criteria for evaluation of brain tumour size and treatment responses were adjusted after introduction of corticosteroids in GBM treatment [35]. Again, new criteria need to be developed now that angiogenesis inhibitors are being explored for the treatment of GBM and, importantly, for other tumours that metastasise to the brain [36-41]. Promising imaging modalities to evaluate anti-angiogenic treated patients include T2-weighted imaging [42], dynamic contrast enhancement imaging [43], apparent diffusion coefficient imaging [44], diffusion tension imaging [45], diffusion-weighted imaging [46], USPIO-enhanced blood volume imaging [34], (multivoxel) MR spectroscopy [42], positron emitting tomography [47,48], and single-photon emission computed tomography nuclear imaging [49]. However, the clinical value of these modalities for the follow-up of GBM patients treated with angiogenesis inhibitors in trials has yet to be confirmed [50].

### Vascularisation and vessel normalisation

The central hypothesis of angiogenesis inhibition as anti-tumour strategy states that tumour growth and expansion are dependent on the ability of the tumour to generate its own supportive vascularisation [51-53]. The actual dependence on angiogenesis is, however, highly dependent on the tumour micro-environment. Indeed, it is an absolute requirement for rapidly proliferating tumours growing in avascular spaces, which encompass most of the subcutaneous murine tumour models (including glioma). Such tumours are indeed very responsive to anti-angiogenic therapy. In clinical tumours (and, in particular, brain cancer), the situation is far more complex. While GBM is considered to be a highly vascular tumour with an extensive angiogenic component driven by VEGF-A (produced by peri-necrotic hypoxic tumour cells), the relative contribution of angiogenic growth aspects is probably low compared to the complete tumour volume. In the angiogenic parts, the quality of the newly formed vessels may be poor [54,55]. Theoretical and preclinical models support the concept that anti-angiogenic treatment leads to vascular normalisation in these areas; immature intratumoural vessels are pruned, vessel hyperpermeability and concomitant tumour interstitial pressure are reduced, and pericyte coverage is restored, resulting in improved tumour vessel perfusion and creating opportunities for combination treatments [56,57]. Vessel normalisation is believed to explain the synergy between bevacizumab and chemotherapy in advanced colorectal cancer patients [9,57]. In confirmation of this concept, it was recently reported that anti-VEGF treatment of subcutaneous melanoma grafts results in vessel normalisation and concomitant better delivery of MRI contrast agents and oxygen to the tumour [58]. In striking contrast, similar treatment of brain tumours (both primary and metastatic) results in reduced contrast-enhanced MRI visibility both in animal models [34,59] and in GBM patients (Figure 1). Thus, functional implications of vessel normalisation may depend on specific features of the tumour micro-environment, enabling an adaptation to circumvent the specific angiogenic blockade [60].

### Invasion and migration

**Figure 2:**
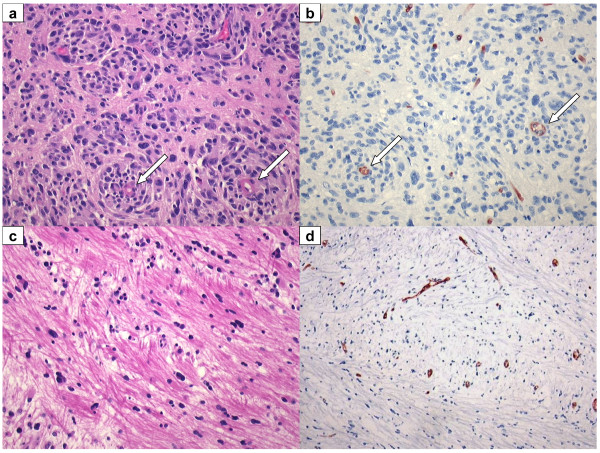
**Recurrent GBM: resection and autopsy material, post bevacizumab**. (**A**, **B**) Recurrent GBM resection material, obtained 6 weeks after last infusion of bevacizumab. Tumour cells co-opt pre-existent vessels with relatively intact BBB (arrows). (**A**) H&E staining 20×. (**B**) Glut-1, BBB marker, 20×. (**C**, **D**) Recurrent GBM: autopsy was performed 10 weeks after the last infusion of bevacizumab. Near tumour sample shows tumour cells invading along white matter tracks. (**C**) H&E 20×. (**D**) Glut-1 BBB marker, 10×.

**Figure 3:**
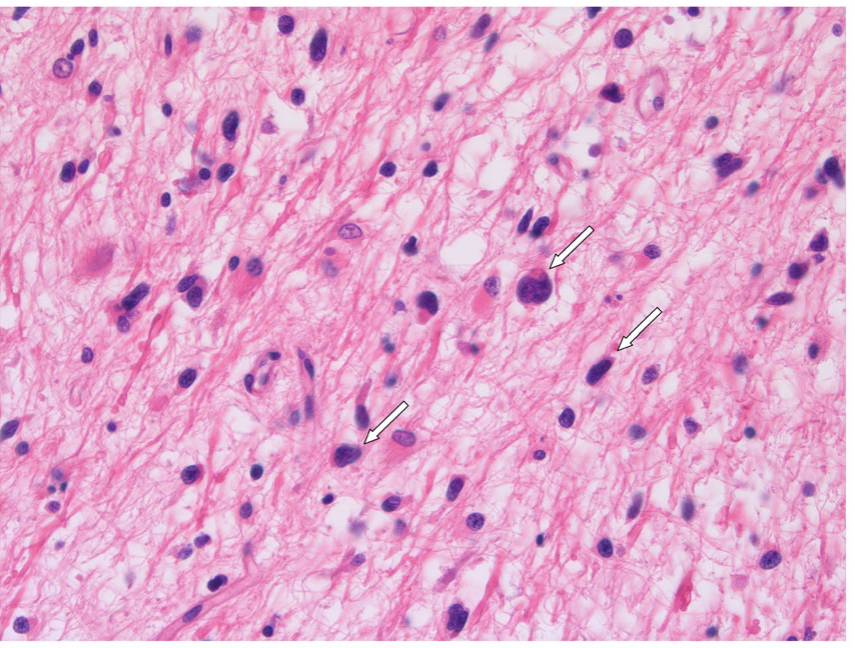
**Recurrent GBM: autopsy material, post bevacizumab**. GBM cells invade almost the whole brain of this recurrent GBM patient (2C, D). H&E stained autopsy specimen of neocortex of the hemisphere opposite to tumour location, 10 weeks after the last infusion of bevacizumab.

Next to the angiogenic rim, in GBM large areas can be identified where viable and proliferating tumour cells invade the surrounding highly vascularised normal tissue. In the cerebrum, blood vessels and white matter tracts are typically located next to each other and form corridors for invading GBM cells [4]. Close to the tumour bulk, glioma cells can be found alongside these vessels and white matter tracts (Figure 2A-D) [61]. Utilisation of these blood vessels for metabolic support (known as vessel co-option), offers proliferating tumour cells an efficient escape mechanism for anti-angiogenic therapy. Indeed, co-opted vessels are refractory to anti-angiogenic treatment [33,56,62-65]. Consequently, even during anti-angiogenic therapy, GBM cells continue to migrate through the adjacent cerebrum for considerable distances as is illustrated by the presence of high densities of tumour cells, disperse throughout the brain of a patient who died 10 weeks after the last dose of bevacizumab (Figure 3). Published clinical data appear in line with recently published preclinical data, showing that VEGF inhibition may promote tumour cell invasiveness, also in other tissues than brain [66-69]. Clinical studies have also shown that bevacizumab may alter the recurrence pattern of malignant gliomas by suppressing contrast-enhancing tumours more effectively than non-enhancing, infiltrative tumours [22,70].

### Blood brain barrier and chemotherapy

Chemotherapeutic drugs are generally not very effective against GBM, partly due to intrinsic resistance [71] but also because of an intact BBB in peripheral parts of the tumour (note the positive staining for BBB-specific endothelial protein glut-1 in Figure 2B,D) that efficiently restricts the distribution of drugs to tumour cells [2]. Even TMZ, which is able to cross the BBB to some extent, probably acts more effectively against tumour cells in more accessible angiogenic parts of the tumour. The synergy of combined anti-angiogenic and chemotherapeutic agents in other malignancies has been partially attributed to normalisation of the tumour vascular bed, as discussed above [53]. Whereas this strategy may work well in tumours outside the brain, in case of GBM normalisation of the tumour vascular bed and reduction of interstitial pressure comes at the expense of restored functionality of the BBB as evidenced by the reduced permeability for Gd-DTPA. The accompanying reduced accessibility of other drugs carries the risk of antagonising the efficacy of such agents; we have shown this in mice bearing orthotopic intracranial tumours treated with TMZ either with or without concomitant anti-angiogenic treatment with vandetanib (ZD6474) [59]. More recently, it was reported that bevacizumab increases efficacy of TMZ [72] or carboplatin [73]. At this moment the cause of this apparent discrepancy remains unclear but effects of timing and dosing of anti-angiogenic therapy and chemotherapy may play a role here. For example, we previously demonstrated that high doses of vandetanib effectively inhibited angiogenesis and restored vessel permeability, whereas low doses did inhibit angiogenesis but left tumour vasculature hyperpermeable [33]. These features should be carefully investigated for clinical GBM too, to improve control of the different therapeutic modalities on tumour behaviour. Meanwhile, the discussion continues as to whether combining anti-angiogenic treatments with other chemotherapies is beneficial or detrimental (or somewhere in-between).

## Conclusions

The relevance of angiogenesis inhibition in the treatment of GBM has been placed in the unique context of malignant brain tumours. Patients may benefit from anti-angiogenic therapy by its reduction of peritumoural oedema and intracranial pressure. Several studies also suggest an anti-tumour effect because of improved response rates and prolonged PFS; however, these data are derived from non-randomised trials with PFS as primary endpoint. Available data on survival prolongation are less robust (phase II) and sometimes even conflicting. High costs and side-effects of angiogenesis inhibitors (e.g. venous and arterial thrombo-embolism and haemorrhage as demonstrated in phase II studies) need to be balanced against advantages related to survival. Therefore, we can only implement angiogenesis inhibitors as standard treatment for patients with GBM when data are available from randomised clinical trials with survival as primary endpoint. Our concerns are not restricted to the limited amount of data on outcome and toxicity, but also arise from preclinical data on the biology of GBM and angiogenesis inhibition.

**Figure 4:**
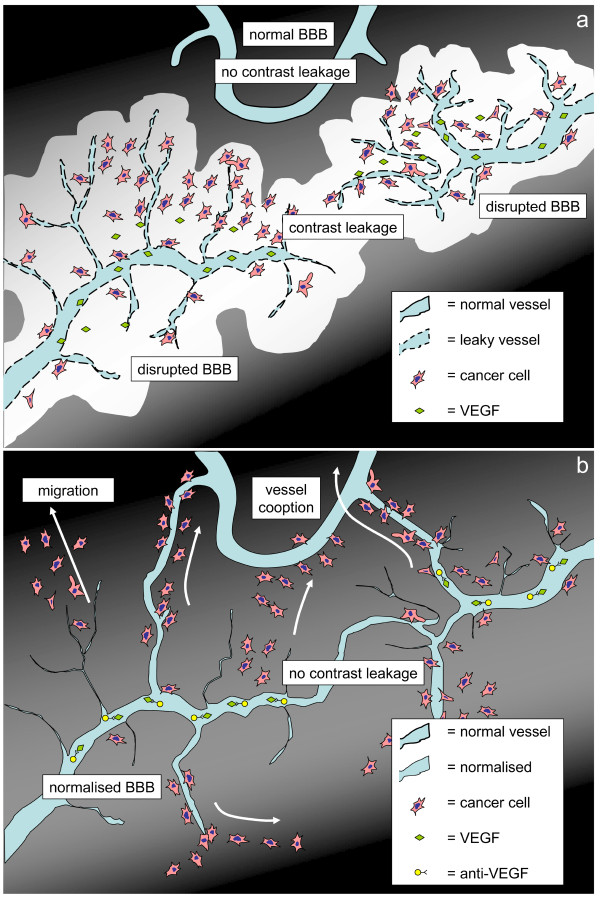
**Schematic drawing: pre-treatment and during treatment**. Schematic drawing of high-grade glioma, pre-treatment (**A**), and with anti-VEGF treatment (**B**). (**A**) Contrast leakage (white) occurs around leaky tumour vessels enhancing the tumour area on MRI. Capillaries in surrounding tissue are not leaky. (**B**) Contrast-enhanced area is strongly reduced under anti-VEGF treatment. Tumour cells migrate furtively into the surrounding tissue and co-opt existing vasculature.

GBM can be roughly separated into an angiogenic component, and an invasive or migratory component. Although this latter component seems inert to anti-angiogenic therapy, it is of major importance for disease progression and survival (Figure 4). Whereas symptoms are tempered by anti-angiogenic treatment, furtive invasion of the disease continues, unrecognised by standard imaging modalities. Moreover, while treatment of other tumour types is improved by combining chemotherapy with anti-angiogenic drugs, inhibiting angiogenesis in GBM may antagonise the efficacy of chemotherapeutic drugs by normalising the BBB function. Although angiogenesis inhibition is of value for symptom reduction in GBM patients, the possible lack of a true anti-tumour effect raises concerns about the place of this type of therapy in the treatment of patients with GBM.

## Summary

1. GBM can be roughly separated into an angiogenic component, and an invasive or migratory component.

2. Although this invasive or migratory component seems inert to anti-angiogenic therapy, it is of major importance for disease progression and survival.

3. Whereas symptoms are tempered by anti-angiogenic treatment, furtive invasion of the disease continues, unrecognised by standard imaging modalities.

4. Moreover, while treatment of other tumour types is improved by combining chemotherapy with anti-angiogenic drugs, inhibiting angiogenesis in GBM may antagonise the efficacy of chemotherapeutic drugs by normalising the BBB function.

5. Although angiogenesis inhibition is of value for symptom reduction in GBM patients, the possible lack of a true anti-tumour effect raises concerns about the place of this type of therapy in the treatment of GBM.

## Competing interests

The authors declare that they have no competing interests.

## Authors' contributions

JJCV, OVT, LJAS, DJR, WPJL and WRVF designed the experiments and the study. JJCV, OVT, AC, WPJL and WRVF collected data for the study. JJCV, OVT, AC, MEVL and WPJL analyzed the data. JJCVV, LJAS, MEVL, DJR and WRVF enrolled patients. JJCV and OVT wrote the first draft of the paper. JJCV, OVT, AC, LJAS, MEVL, DJR, WPJL and WRVF contributed to the writing of the paper. All authors read and approved the final manuscript.

## Pre-publication history

The pre-publication history for this paper can be accessed here:

http://www.biomedcentral.com/1471-2407/9/444/prepub
